# A case report of eosinophilia associated with risperidone withdrawl in a patient with schizophrenia

**DOI:** 10.1192/j.eurpsy.2022.1837

**Published:** 2022-09-01

**Authors:** E. Giourou, A. Theodoropoulou, P. Batzikosta, O. Prodromaki, E. Georgila, P. Gourzis

**Affiliations:** General University Hospital of Patras, Greece, Department Of Psychiatry, Patras, Greece

**Keywords:** risperidone withdrawl, eosinophilia

## Abstract

**Introduction:**

Risperidone, a second generation antipsychotic, shows high affinity with serotoninergic and dopaminergic D2 receptors, but also adrenergic and H1 histaminergic receptors. Previous studies have shown an increase in eosinophile count associated with the second-generation antipsychotics through the histaminergic path.

**Objectives:**

The presentation of a case in which eosinophilia was associated with risperidone withdrawl which has not been described so far.

**Methods:**

A 46-year-old woman with schizophrenia diagnosed at the age of 22 was admitted in our inpatient psychiatric clinic with psychotic symptoms relapse after she voluntarily discontinued risperidone. The patient was fully evaluated with full laboratory tests, a brain CT scan, EEG and her medical and psychiatric histories were recorded.

**Results:**

Risperidone was reinitiated but due to the persistence of symptoms it was switched to clozapine which lead to full remission. It was observed though, that while gradually decreasing risperidone dosage (Figure 1.), eosinophile count was raising and it was normalized after complete discontinuation. Eosinophilia was also present in other instances that the patient discontinued taking risperidone according to her personal history. Other causes of eosinophilia (allergic, inflammatory) were fully excluded.

**Figure:**
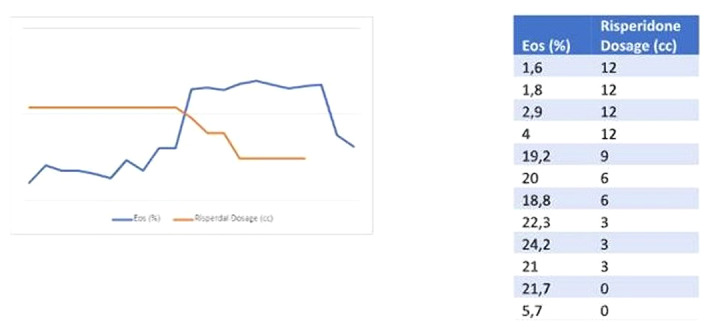

**Conclusions:**

Risperidone discontinuation could lead to an elevated eosinophile count. There is limited research in this topic and it is yet to be clarified whether the elevation is due to stopping one antipsychotic or switching between two different antipsychotics. It is important to run laboratory tests regularly with every treatment modification.

**Disclosure:**

No significant relationships.

